# Common evolutionary trajectory of short life-cycle in Brassicaceae ruderal weeds

**DOI:** 10.1038/s41467-023-35966-7

**Published:** 2023-01-18

**Authors:** Ling-Zi Li, Zhou-Geng Xu, Tian-Gen Chang, Long Wang, Heng Kang, Dong Zhai, Lu-Yi Zhang, Peng Zhang, Hongtao Liu, Xin-Guang Zhu, Jia-Wei Wang

**Affiliations:** 1 National Key Laboratory of Plant Molecular Genetics (NKLPMG), CAS Center for Excellence in Molecular Plant Sciences (CEMPS), Institute of Plant Physiology and Ecology (SIPPE), Chinese Academy of Sciences (CAS), Shanghai, 200032 China; 2 University of Chinese Academy of Sciences, Shanghai, 200032 China; 3 Department of Computer Science and Technology, Nanjing University, Nanjing, 210093 China; 4 School of Life Science and Technology, ShanghaiTech University, Shanghai, 201210 China

**Keywords:** Natural variation in plants, Plant development, Genetic variation, Evolutionary genetics

## Abstract

Weed species are detrimental to crop yield. An understanding of how weeds originate and adapt to field environments is needed for successful crop management and reduction of herbicide use. Although early flowering is one of the weed trait syndromes that enable ruderal weeds to overcome frequent disturbances, the underlying genetic basis is poorly understood. Here, we establish *Cardamine occulta* as a model to study weed ruderality. By genome assembly and QTL mapping, we identify impairment of the vernalization response regulator gene *FLC* and a subsequent dominant mutation in the blue-light receptor gene *CRY2* as genetic drivers for the establishment of short life cycle in ruderal weeds. Population genomics study further suggests that the mutations in these two genes enable individuals to overcome human disturbances through early deposition of seeds into the soil seed bank and quickly dominate local populations, thereby facilitating their spread in East China. Notably, functionally equivalent dominant mutations in *CRY2* are shared by another weed species, *Rorippa palustris*, suggesting a common evolutionary trajectory of early flowering in ruderal weeds in Brassicaceae.

## Abstract (web summary)

Understanding origin and adaptation of weeds is important for their management. Here, via genome assembly, population genomics, and QTL mapping, the authors establish *Cardamine occulta* as a model to study weed ruderality and show *FLC* and *CRY2* as genetic drivers for the establishment of short life cycle.

## Introduction

Weed species are among the greatest pests of agriculture, causing ~10% worldwide reduction in crop productivity each year^1–3^. An understanding of how weeds originate and adapt to field environments is needed for successful crop management and reduction of herbicide use^4^. Notably, human-crop-weed interactions have emerged as a fascinating system to understand the impact of human activities on ecological and evolutionary dynamics^5^. Moreover, better knowledge of the innovations behind the adaptation and rapid evolution of weed species could help us to uncover basic principles related to the origin and divergence of new species.

Based on their genetic relationship to crops, agricultural weeds (also known as arable weeds) can be mainly divided into two classes, namely weedy crop relatives and non-crop relatives^6^. Agricultural weed syndrome refers to the traits that enable weeds to survive and thrive and become abundant and difficult to eradicate within areas of human disturbance^7^. In general, these adaptive traits include but are not limited to a short life cycle, high nutrient use efficiency, optimal length of seed dormancy, efficient seed dispersal, herbicide resistance, and crop mimicry^8^.

There are three main paths through which a plant species can become a weed, namely crop-wild hybridization, crop de-domestication, and invasion of field by wild species^6,8–11^. For example, two weedy sorghums (i.e., sudangrass and shattercane, *Sorghum bicolor* ssp. *drummondii*) evolved through hybridization between cultivated and wild sorghum^12^, whereas weedy rice (*Oryza sativa* *f.* *spontanea*) originated from de-domestication and feralization of cultivated ancestors^13–18^. By contrast, barnyardgrass (*Echinochloa crus-galli*), a notorious non-crop relative weed in paddy fields, evolved through human selection on Vavilovian mimicry^19,20^. Despite these achievements in characterizing the evolution of weedy species, the genetic basis and functional properties of agricultural weed syndrome are still largely unknown^21^.

Grime’s CSR model predicts that plants have three major life-history strategies and can be classified as competitors (C), stress-tolerators (S), and ruderals (R)^22,23^. Ruderality is a typical feature of weeds. To adapt to low stress, high-disturbance (the partial or total destruction of the plant biomass during the growing seasons by the activities of herbivores, pathogens, man, and environment such as wind damage, frosts, desiccation, and fire) regimes, ruderals allocate resources mainly to seed reproduction and are often annuals or short-lived perennials. Common characteristics of ruderal species include short life-cycle, a high relative growth rate, abundant seed production, and a short stature with minimal lateral expansion^6,10,24^. However, due to the lack of suitable plant models, the genes responsible for weed ruderality are currently unknown.

**Fig. 1:**
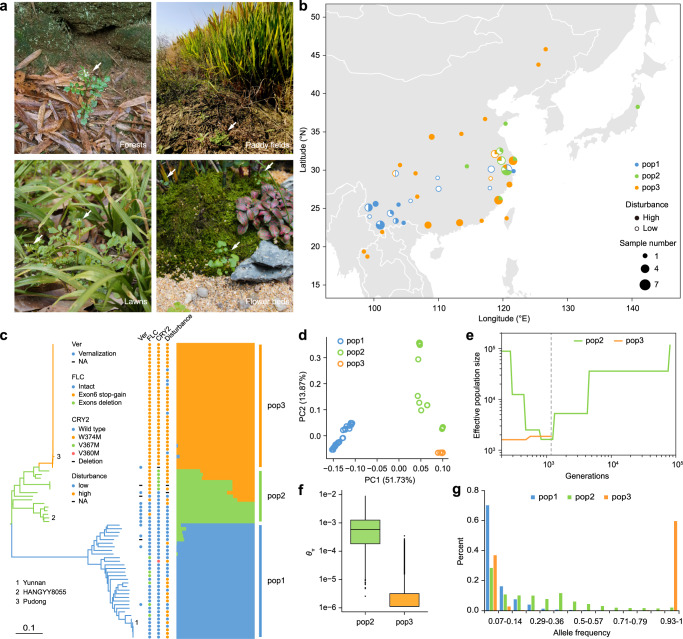
The population structure of *C. occulta* in East Asia. **a** Representative *C. occulta* plants growing in forests, paddy fields, lawns, and flower beds. The white arrows point to wild *C. occulta* plants. **b** Distribution of the *C. occulta* accessions used in this study. The area of each pi represents the sample size in each site, and the colors indicate *C. occulta* subgroups. The filled circles and the open circles represent the high-disturbance and low-disturbance habitats, respectively. **c** A neighbor-joining tree of 82 *C. occulta* accessions constructed using whole-genome SNP data. Branch colors denote subgroups. Three representative accessions, Yunnan, HANGYY8055, and Pudong, are marked with 1, 2, and 3, respectively. The colors of the dots to the right of the tree indicate vernalization requirement (Ver), haplotypes of *FLC* and *CRY2*, and degree of habitat disturbance of each accession. Short black lines, data not available. The ADMIXTURE results at *k* = 3, which had the lowest cross-validation error, are shown on the right. Subgroups (pop1, pop2, and pop3) are indicated by the colored bars. **d** PCA of 82 *C. occulta* accessions. The proportion of the variance explained is 51.73% for PC1 and 13.87% for PC2. **e** Population history inferred by SMC++. Per-generation mutation rate was assumed to be 7.1 × 10^−9^. The dashed vertical line indicates the time of divergence (nearly 1000 generations ago). **f** Nucleotide diversity (*θ*π per 50 kb) of pop2 (*n* = 15 accessions) and pop3 (*n* = 35 accessions). Nucleotide diversity (*π*) values were calculated using the whole-genome SNP data with a window size of 50 kb and a step size of 20 kb. The black lines in the box represent the median values and the lower and upper hinges correspond to the first and third quartiles. The upper and lower whiskers extend from the hinge to the values > or <1.5 × interquartile range from the hinge. **g** The frequency spectrum of *C. occulta* populations. The site frequency spectrum was estimated for 30, 15, and 30 individuals from pop1, pop2, and pop3, respectively, using the whole-genome SNP data.

With the rapid development in genome sequencing technology, population genomics, and pan-genome-based association studies have emerged as valuable approaches to identify key genetic determinants underlying weediness^8,25–29^. *Cardamine occulta* (2n = 8x = 64) is an annual, self-pollinated, octoploid ruderal weed that most likely originated in Eastern Asia, but it has also been introduced to other continents including Europe^30–33^ (Fig. 1a). The completion of the *Cardamine hirsuta* reference genome and the fact that *C. occulta* is a close relative of the model plant *Arabidopsis thaliana* enable us to use *C. occulta* as a model to characterize the genetic and molecular basis for weed ruderality in Brassicaceae^34–37^.

Using genome assembly and QTL mapping, we show here that sequential mutations in the vernalization response regulator gene *FLOWERING LOCUS C* (*FLC*) and blue-light receptor gene *CRYPTOCHROME2* (*CRY2*) were critical steps during the evolution of short life-cycle in *C. occulta*. Through a population genomics approach, we further demonstrate that individuals carrying these two mutations can flower early under a broad range of photoperiod conditions and overcome human disturbance through early deposition of seeds into the soil seed bank, thereby expanding their distribution range in East China. Moreover, using *Rorippa palustris* as a second genetic model, we find that this evolutionary trajectory may have been followed by other ruderal weeds in Brassicaceae.

## Results

### The collection of *C. occulta* accessions and genome assembly and annotation

We collected 82 *C. occulta* accessions across China, Japan, and Thailand (Fig. 1a, b). All the accessions are octoploid and belong to the same species as indicated by phenotyping (Supplementary Fig. 1a), flow cytometry assay (Supplementary Fig. 1b), and genome re-sequencing (see below, Supplementary Data 1). Growth habitats of *C. occulta* include roadsides, flower beds, paddy fields, forests, and mountains (Fig. 1a; Supplementary Data 1). Consistent with the notion that *C. occulta* is a hygrophile, no accessions were found in the arid regions in Northwest China (Fig. 1b). We assembled the genome of an accession collected from Yunnan Province, China (Yunnan accession) using Oxford Nanopore Technologies (ONT) sequencing data combined with Illumina next-generation sequencing data and Hi-C chromatin interaction maps. In total, we generated 93.79 Gb of ONT long reads, 69.46 Gb of short reads, and 100.32 Gb of Hi-C data. We de novo assembled the ONT long reads into 1118 high-quality contigs using Canu assembler and NextPolish^38,39^. The resulting genome assembly of *C. occulta* was 680.6 Mb with a contig N50 length of 4.37 Mb. We identified allelic contigs based on syntenic genes shared by *C. occulta* and *C. hirsuta* and used the ALLHIC pipeline to phase and scaffold the contigs^40^. As a result, 32 pseudo-chromosomes consisting of 8 homologous groups with four sets of monoploid chromosomes were assembled (99.4% of the assembly) (Supplementary Fig. 1c). The statistics of the *C. occulta* genome are given in Supplementary Data 2.

Using a combination of ab initio-based, homology-based, and transcriptome-based approaches, 101,390 protein-coding genes were predicted in the *C. occulta* genome. By re-sequencing all 82 accessions, we generated 1.1 trillion base pairs of sequencing data with an average coverage depth of 17.75-fold, ranging from 12.24- to 38.81-fold, based on the reference Yunnan genome (Supplementary Data 1). We obtained 4.7 million high-quality single-nucleotide polymorphisms (SNPs), of which 997,906 were located in coding regions, causing 846,750 nonsynonymous mutations, 193,549 synonymous mutations, 776 start codon changes, and 10,173 stop codon changes (Supplementary Data 3).

### Population structure analysis

A whole-genome neighbor-joining tree between the *C. occulta* accessions was inferred on the basis of the SNPs across 82 samples. As shown in Fig.1c, all 82 *C. occulta* accessions can be divided into three subgroups, namely pop1, pop2, and pop3. Pop1 and pop2 exhibit a close relationship and shared ancestry (Fig. 1c, d), suggesting that they are derived from the same ancestral population. In agreement with principal component analysis (PCA), pop3 clusters as a single clade with low segregating variation within the clade (Fig. 1c, d). The topology supported that pop3 is likely to be derived from pop2. A substantial proportion of the SNPs identified in pop3 were shared with pop2, reflecting its role as a genetic resource to pop3 (Supplementary Fig. 1d). We estimated the divergence time of pop2 and pop3 jointly with population size histories using the 2-population clean-split model implemented in SMC++^41^. The analysis suggests that pop2 experienced a steep decline in effective population size (*Ne*) (Fig. 1e). Pop3 diverged from pop2 ~1000 years ago (assuming one generation per year) and had a low *Ne* thereafter. Intriguingly, this divergence time largely overlaps with the time of origin of the rice weed *E. crus-galli*^19^.

Assessments of genome-wide nucleotide diversity (*π*) indicated that pop3 accessions harbor lower genetic diversity than pop2 accessions (Fig. 1f), suggesting a possible bottleneck during the divergence of pop3 from pop2. Consistent with this, pop3 had the lowest linkage disequilibrium decay rate and a U‐shaped site‐frequency spectrum (Fig. 1g; Supplementary Fig. 1e). Moreover, most SNPs in pop2 were fixed in pop3 (Supplementary Fig. 1f).

### Adaptation of pop3 to high-disturbance environments

**Fig. 2:**
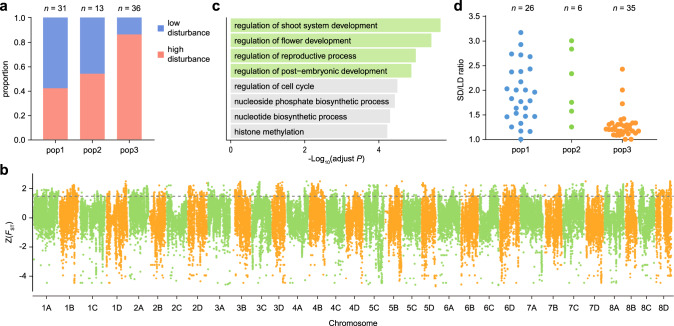
Adaptation of pop3 to high-disturbance environments. **a** The proportion of plants from three populations in low-disturbance areas (i.e., forests and mountains) and high-disturbance areas (i.e., paddy fields, flower beds, and roadsides). See also Supplementary Data 1. **b** *F*_ST_ values of pop2 and pop3 accessions across the whole genome. The gray dashed line represents the top 5% threshold. **c** GO term analyses of the genes within the top 5% *F*_ST_ regions. The top eight enriched GO biological processes are shown. The biological processes shaded in green are associated with the transition from vegetative-to reproductive growth. See also Supplementary Data 4 and Supplementary Figs. 2a, b. **d** Flowering time of *C. occulta* accessions. The SD/LD ratio was calculated by dividing the median number of total leaves when plants started to bolt in SD by the median number of total leaves when plants started to bolt in LD. Plants were grown in a growth chamber. The accessions are grouped by subgroups, and each dot represents an accession. See also Supplementary Fig. 2c. The source data underlying Fig. 2d are provided as a Source Data file.

It has been reported that *C. occulta*, as an invasive weed species, is better able to adapt to environments with a high degree of disturbance related to human activities than its related species^30,31^. We, therefore, classified the growth habitats of *C. occulta* accessions into two categories, namely high-disturbance (e.g., roadsides, flower beds, and paddy fields) and low-disturbance (e.g., forests and mountains) areas. Intriguingly, while pop1 and pop2 accessions frequently reside in low-disturbance areas, most pop3 plants are found in high-disturbance areas (Figs. 1b, 2a). In line with this finding, analyses of geographic distribution revealed that pop3 plants are widely distributed in East China, in contrast to the more restricted distributions of pop1 and pop2 (Fig. 1b). Since pop3 exhibits the lowest genetic diversity (Fig. 1), these results collectively imply that pop3 developed ruderal growth habit characteristics.

To identify the genomic regions with signatures of adaptive differentiation between pop3 and its inferred ancestor pop2, genomic scans of differentiation (*F*_ST_) were performed (Fig. 2b). The genes in the top 5% range were selected for Gene Ontology (GO). In agreement with the predictions of Grime’s CSR model, there was significant enrichment in pathways related to the vegetative-to-reproductive growth transition (Fig. 2c; Supplementary Data 4). Similar GO terms were also identified by SNP2GO (Supplementary Figs. 2a,b), a program to test for the overrepresentation of candidate SNPs in biological pathways^42^. These results suggest that variation in flowering time genes might contribute to the adaptation and widespread distribution of pop3.

Flowering time is regulated by environmental cues. The climate in the regions where *C. occulta* accessions are distributed is highly diverse. Notably, the early-flowering pop3 plants have a wide distribution range spanning from the tropical, subtropical, warm temperate, to mid-temperate zones (18.7 to 45.8°N, Fig. 1b). The annual daylength ranges from 8–16 hours^43^. Five flowering time pathways, namely the age, autonomous, photoperiod, gibberellin, and vernalization pathways, have been extensively studied in *Arabidopsis*^44–48^. We found that some individual pop1 and pop2 accessions exhibit a vernalization requirement, whereas nearly all the pop3 plants are capable of flowering without long-term cold treatment (Fig. 1c; Supplementary Data 1). Thus, these findings show that the vernalization response is an ancestral trait of *C. occulta* and that loss of the vernalization requirement contributed to the establishment of the ruderal growth habit of pop3.

We next surveyed the flowering time of all accessions. Ideally, this experiment should be carried out in the field. However, due to the practical difficulties, we only measured the flowering time in the growth chamber under both long-day (LD, 16-h light/8-h dark) and short day (SD, 8-h light/16-h dark) conditions, which represent the maximum and minimum daylength in the habitats of *C. occulta*. Using the ratio of the total number of leaves when the plants started to flower (bolt) in SD to that in LD as an index, we found that the majority of pop1 and pop2 accessions are early-flowering in LD, while most pop3 plants are photoperiod-insensitive (Fig. 2d; Supplementary Fig. 2c). Therefore, the switch from LD to day-neutral flowering likely served as the second critical step during the evolution of the ruderal growth habit in *C. occulta*. The short life-cycle of pop3 under a broad range of photoperiod conditions improves its adaptability to human disturbance, thereby expanding its distribution range.

### Natural variation in *FLC* underlies the loss of the vernalization requirement in pop2 and pop3

**Fig. 3:**
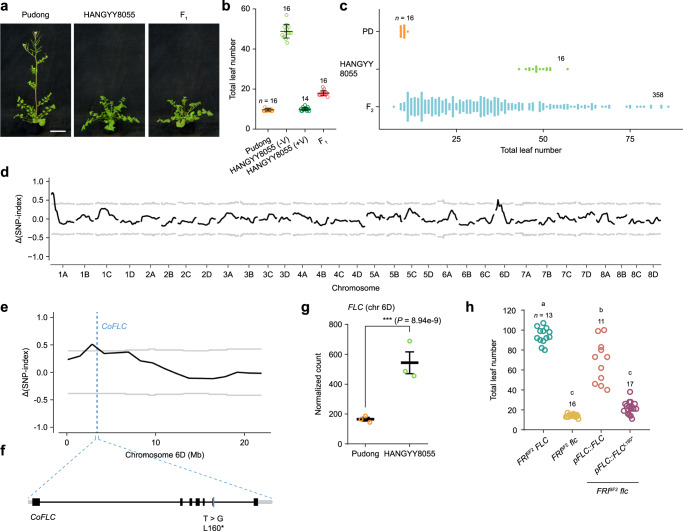
Identification of *FLC* as the gene responsible for the loss of the vernalization requirement in pop3. **a**, **b** Flowering phenotypes (**a**) and flowering time (**b**) of the Pudong and HANGYY8055 accessions and Pudong × HANGYY8055 F_1_ plants grown in LD in a growth chamber. +/−V, with or without vernalization treatment. Scale bar in (**a**) 5 cm. **c** Flowering time of the Pudong and HANGYY8055 accessions and plants in the Pudong × HANGYY8055 F_2_ segregating population in LD. **d** Genome-wide Δ(SNP index) plot from BSA of the F_2_ segregating population derived from a cross between Pudong and HANGYY8055. The black lines indicate tricube-smoothed Δ(SNP index), and gray lines represent the corresponding two-sided 99% confidence intervals. **e** The Δ(SNP index) plot of Chr 6D from BSA of the F_2_ segregating population derived from a cross between Pudong and HANGYY8055. The black line indicates tricube-smoothed Δ(SNP index), and gray lines mark the corresponding two-sided 99% confidence intervals. The blue dotted line indicates the location of *C. occulta FLC* (*CoFLC*) on Chr 6D. **f** The gene structure of *CoFLC* and the location of the mutation (blue line). Black boxes, gray boxes, and black lines represent exons, untranslated regions (UTRs), and introns, respectively. **g** Analysis of the transcript levels of *CoFLC* genes on Chr 6D in the Pudong and HANGYY8055 accessions by RNA-seq. Normalized counts and adjusted *P* values were both analyzed by DESeq2. The *P* values attained by the two-sided Wald test were corrected for multiple testing using the Benjamini and Hochberg methods. Data are mean ± s.e. of three biological replicates (open circle). ns, not significant. **h** Flowering time of *Arabidopsis FRI*^*SF2*^ *FLC*, *FRI*^*SF2*^ *flc,* and T_1_ transgenic lines in LD. We generated an allele of *Arabidopsis FLC* (*FLC*^L160*^) that mimics the mutation in *FLC* allele of pop3. Plants were grown in a growth chamber. Letters indicate significant differences as determined by ordinary one-way ANOVA. Error bars in **b** denote s.d. The number of examined plants (*n*) is given. The centers of the error bars represent the mean values. The source data underlying Fig. 3b, c, and h are provided as a Source Data file.

To understand the genetic basis for the loss of vernalization response in *C. occulta*, we crossed two representative accessions, Pudong (pop3, an accession collected in the Pudong District of Shanghai) and HANGYY8055 (pop2) (Fig. 3a). Compared with Pudong, the HANGYY8055 accession flowered late in LD (Fig. 3b) and this late-flowering phenotype could be largely reversed by vernalization (Fig. 3b; Supplementary Data 1). The Pudong × HANGYY8055 F_1_ plants flowered early under LD conditions without vernalization (Fig. 3b). To identify the casual gene, we prepared two bulked DNA samples of the F_2_ population, one representing early-flowering (*n* = 43) individuals and the other late-flowering (*n* = 46) individuals (Fig. 3c), and performed next-generation sequencing. The quantitative trait loci (QTLs) controlling flowering time were inferred by QTL-seq^49^. One candidate region located in the 2.113–3.846 Mb interval on chromosome 6D (Chr 6D) was identified (Fig. 3d; Supplementary Data 5). Within this region, we found *FLC*, which encodes a repressor of flowering that confers a requirement for vernalization^44,50,51^ (Fig. 3e).

Genome re-sequencing revealed the *FLC* copy on Chr 6D in Pudong possessed a nonsense mutation at position 160 in the sixth exon (leucine to stop codon, hereafter referred to *FLC*^L160*^, Fig. 3f; Supplementary Fig. 2d). Interestingly, a haplotype similar to *FLC*^L160*^ has been identified in *Arabidopsis*^52^. Transcriptome sequencing indicated that the expression of the *FLC*^L160*^ allele in the Pudong accession was significantly lower than that of the *FLC* allele in HANGYY8055 (Fig. 3g). By contrast, the transcript levels of other flowering time genes within the candidate region were largely unaffected (Supplementary Fig. 2e). Moreover, transgenic studies in *Arabidopsis* confirmed that the *FLC*^L160*^ allele is functionally impaired (Fig. 3h).

The survey of the *FLC* genomic sequences revealed that the *FLC* copy on Chr 6 C lacked exons 2, 3, and 4 (hereafter referred to *FLC*^*exon-*^) in all the *C. occulta* accessions (Supplementary Fig. 2f). The expression level of the *FLC* copy on Chr 6B was low in general (Supplementary Fig. 2f). Some pop2 and all the pop3 accessions including Pudong harbor the *FLC*^L160*^ mutation on Chr 6D (Fig. 1c). Taken together, the above results are consistent with a previous report that FLC acts as a semi-dominant repressor of flowering^53^. The simultaneous impairment of two *FLC* copies (Chr 6 C and Chr 6D) in Pudong, together with a lowly expressed *FLC* copy on Chr 6B, results in the downregulation of *FLC* activity, which contributes to the loss of the vernalization response. Notably, the *FLC*^L160*^ mutation arises in pop2 and is fixed in pop3.

### Dominant mutation of *CRY2* is responsible for early flowering in short days

**Fig. 4:**
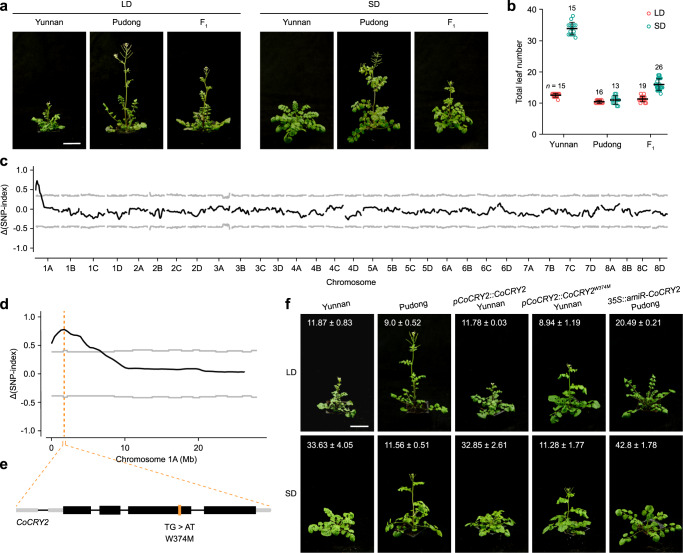
Identification of *CRY2* as the gene responsible for early-flowering in short days. **a**, **b** Flowering phenotypes (**a**) and flowering time (**b**) of the Yunnan and Pudong accessions and Yunnan × Pudong F_1_ plants grown under LD or SD conditions in a growth chamber. Error bars denote s.d. The number of examined plants (*n*) is given. The centers of the error bars represent the mean values. **c** Genome-wide Δ(SNP index) plot from BSA of the F_2_ segregating population derived from a cross between Pudong and Yunnan. The black lines indicate tricube-smoothed Δ(SNP index), and the gray lines indicate corresponding two-sided 99% confidence intervals. **d** Δ(SNP index) plot of Chr 1 A from BSA of the F_2_ segregating population derived from a cross between Pudong and Yunnan. The black line indicates tricube-smoothed Δ(SNP index), and gray lines mark the corresponding two-sided 99% confidence intervals. The orange dotted line indicates the location of *C. occulta CRY2* (*CoCRY2*). **e** Gene structure of *CoCRY2*. Black boxes, gray boxes, and black lines represent exons, untranslated regions, and introns, respectively. The mutation site (W374M) is labeled with an orange line. **f** Flowering time of the Yunnan and Pudong accessions and transgenic lines in LD (upper panels) or SD (lower panels) in a growth chamber. One representative plant for each genotype is shown. The flowering time is given as mean ±s.d. The number of examined plants (*n*) is given in Supplementary Fig. 3g. Scale bar in (**a**, **f**), 5 cm. The source data underlying Fig. 4b, f are provided as Source Data file.

To understand the genetic basis for the natural variation in photoperiod sensitivity of pop3, we crossed two representative accessions, Yunnan (pop1) and Pudong (pop3). As Pudong, the Yunnan accession does not require vernalization to flower (Fig. 4a, b). The Yunnan × Pudong F_1_ plants flowered early in SD, implying that the day-neutral phenotype of Pudong is also caused by a semi-dominant mutation(s) (Fig. 4a, b). To clone the causal gene, we performed next-generation sequencing-based bulk segregant analysis (BSA) of the F_2_ population. Two bulked DNA samples were prepared from early-flowering (*n* = 50) and late-flowering plants (*n* = 49) in SD (Supplementary Fig. 3a), and QTLs were inferred by QTL-seq^49^. One candidate region was identified in the 0.001–6.768 Mb interval on chromosome 1 A (Fig. 4c, d; Supplementary Data 6). This region contains a total of 16 flowering time genes, six of which are involved in the photoperiodic pathway, according to the FLOR-ID database^54^. Expression analyses revealed that there was no significant difference in the expression levels of these genes between Pudong and Yunnan (Supplementary Fig. 3b).

The blue-light receptor gene *CRY2* is a promising candidate among the six photoperiodic pathway genes^46,55^. Previous reports have shown that, upon activation by blue-light, CRY2 promotes flowering by inducing expression of the florigen gene *FLOWERING LOCUS T* (*FT*) by stabilizing CONSTANS (CO) or interacting with bHLH transcription factors including CRYPTOCHROME-INTERACTING BASIC-HELIX-LOOP-HELIX1 (CIB1)^56–61^. Indeed, genome re-sequencing revealed a nonsynonymous mutation at position 374 (tryptophan to methionine substitution, CRY2^W374M^) in the *CRY2* coding region in both Pudong and the early-flowering F_2_ individuals (Fig. 4e; Supplementary Fig. 3c). Among the four copies of the *CRY2* gene in the Pudong genome, only one copy was *CRY2*^*W374M*^, while other three copies were *CRY2*^*WT*^ (the wild-type *CRY2* allele). Transcriptome sequencing indicated that the transcript levels of the *CRY2*^*W374M*^ and *CRY2*^*WT*^ alleles are largely comparable (Supplementary Fig. 3b).

The W374M mutation affects one of three evolutionarily conserved tryptophan residues (W321, W374, and W397) known as the “Trp triad” and leads to constitutive activation of CRY2^62^, suggesting that CRY2^W374M^ might represent a constitutively active form of CRY2. Consistent with this hypothesis, yeast two-hybrid (Y2H) assays revealed that CRY2^W374M^ interacted with CIB1 and SPA1 (SUPPRESSOR OF PHYA-105 1, another well-known CRY2-interacting protein) irrespective of light conditions (Supplementary Fig. 3d)^60^. Moreover, expression analyses found that *FT* transcripts were barely detectable in Yunnan but highly abundant in Pudong at zeitgeber time (ZT) 16 in SD (Supplementary Fig. 3e,3f). Furthermore, the introduction of CRY2^W374M^ into the Yunnan accession and *Arabidopsis* led to an early-flowering phenotype in SD, whereas silencing of *CRY2* by an artificial microRNA resulted in late-flowering of the Pudong accession in both LD and SD (Fig. 4f; Supplementary Fig. 3g; see also Fig. 6b below). Taken together, we conclude that *CRY2* is a major causal gene for the day-neutral phenotype of the Pudong accession and that the W374M mutation leads to constitutive activation of CRY2.

### Association of *CRY2* dominant mutations with the adaptability of pop3 to high-disturbance environments

**Fig. 5:**
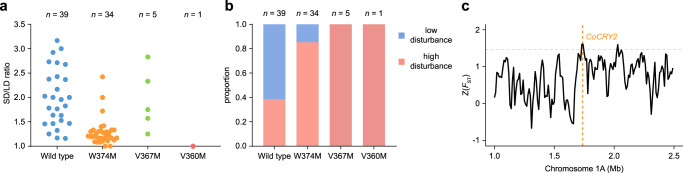
*CRY2*^*W374M*^ contributes to the adaptation of pop3 to high-disturbance environments. **a** Dominant mutations in *CRY2* contribute to early-flowering in SD. The SD/LD ratio was calculated by dividing the median number of total leaves when plants started to bolt in SD by the median number of total leaves when plants started to bolt in LD. The 79 *C. occulta* accessions are grouped by *CRY2* haplotypes. Each dot represents an accession. **b** The proportion of the *C. occulta* accessions with dominant mutations in *CRY2* in high-disturbance and low-disturbance areas. The number of accessions for each *CoCRY2* haplotype is given at the top. **c** Genome-wide *F*_ST_ values in pop2 and pop3 across the 1.5 Mb regions spanning *CoCRY2* on chromosome 1 A. The gray dashed line represents the top 5% threshold. The orange dashed line marks the position of *CoCRY2*. The source data underlying Fig. 5a are provided as a Source Data file.

To ascertain whether the mutation in *CRY2* contributes to the adaptability of pop3 accessions to high-disturbance environments, we surveyed the *CRY2* genomic sequences in all the *C. occulta* accessions. All the pop3 accessions carry the CRY2^W374M^ mutation, whereas six pop2 accessions (40%) have a substitution of valine for methionine at position 367 (CRY2^V367M^) (Fig. 1c; Supplementary Fig. 4a; Supplementary Data 1). Intriguingly, the CRY2^V367M^ mutation is also found in the *Arabidopsis* and contributes to the photoperiod-insensitive phenotype^63^. It should be noted that, among all the sequenced 1135 *Arabidopsis* genomes, the CRY2^V367M^ mutation is only identified in the Cvi-0 accession which lives on the Cape Verde Islands (https://1001genomes.org/)^64^. The Cvi accession is genetically divergent from other accessions, and referred to as a relict^65,66^. Therefore, the ecological significance of the CRY2^V367M^ mutation in *Arabidopsis* has not yet been explored. We also identified the CRY2^V360M^ mutation (valine for methionine at position 360) in a pop1 accession (Fig. 1c; Supplementary Fig. 4b). Y2H and transgenic plant assays revealed that CRY2^V360M^, like CRY2^W374M^ and CRY2^V367M^, is constitutively active (see below). Flowering time measurement revealed a clear correlation between dominant mutations in *CRY2* and early-flowering in SD (Fig. 5a).

Analyses of geographic distribution demonstrated that the *C. occulta* accessions from low-disturbance areas often harbor the CRY2^WT^ allele, while those with the CRY2^W374M^, CRY2^V367M^, or CRY2^V360M^ allele are mainly observed in high-disturbance areas and are widely distributed in East China (Fig. 5b; Supplementary Fig. 4c; Supplementary Data 1). Moreover, we identified a signature of population differentiation in the *CRY2* gene, as indicated by the *F*_ST_ values across the 1.5 Mb genomic region spanning *CRY2* on chromosome 1 A (Figs. 2b, 5c; Supplementary Fig. 4d). Thus, these results collectively indicate that natural variation in *CRY2* is highly associated with the ruderal growth habit of *C. occulta*.

The above results collectively suggest a step-wise evolution of *FLC* and *CRY2* as the major driver for the early-flowering phenotype in the ruderal population (pop3) in *C. occulta*. The simultaneous impairment of two *FLC* copies results in the loss of the vernalization requirement. The dominant mutation in *CRY2* further accelerates flowering, thereby improving its adaptability to human disturbance and expanding its distribution range. A plausible explanation for why *C. occulta* accessions with a constitutively active form of CRY2 have an evolutionary advantage in high-disturbance environments is that these early-flowering individuals can adapt to artificial disturbances (e.g., the clearance of all living plants on the ground) through early deposition of seeds into the soil seed bank, whereas the number of individuals with wild-type *CRY2* genes drops significantly upon artificial disturbances.

### Dominant mutations in CRY2 might serve as a common genetic basis for short life-cycle in Brassicaceae ruderal weeds

**Fig. 6:**
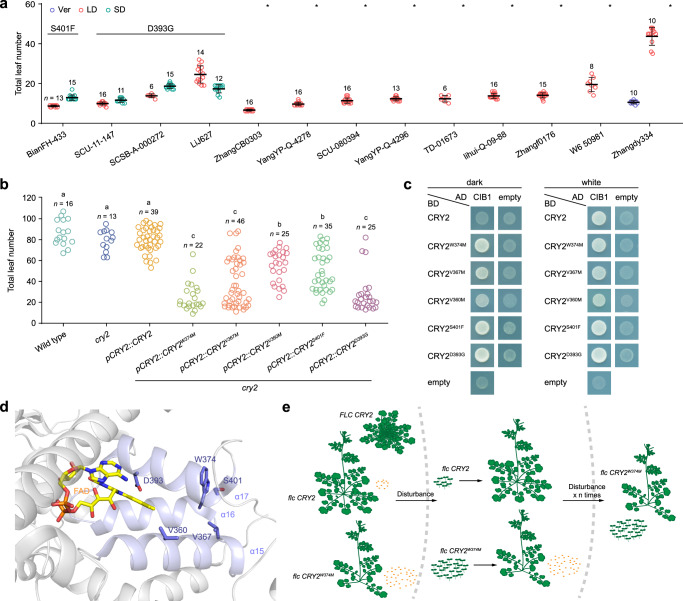
Dominant mutations in *CRY2* might serve as a common genetic basis for ruderality in Brassicaceae. **a** Flowering time of *R. palustris* accessions. Plants were grown under LD, SD, or vernalization conditions in a growth chamber. The total number of leaves when plants started to bolt was counted. Asterisks indicate plants that did not flower within 1 year. The number of examined plants (*n*) is given. Error bars denote s.d. The centers of the error bars represent the mean values. **b** Flowering time of wild-type (Col-0), *cry2*, and T_1_ transgenic *Arabidopsis* lines. Plants were grown in a growth chamber under SD conditions. The different versions of *CRY2* (*CRY2*^*W374M*^, *CRY2*^*W367M*^, *CRY2*^*V360M*^, *CRY2*^*S401F*^, and *CRY2*^*D393G*^) were expressed from the *Arabidopsis CRY2* promoter. The *CRY2*^*W374M*^, *CRY2*^*V367M*^, and *CRY2*^*V360M*^ haplotypes were identified in *C. occulta* accessions (Fig. 1c), whereas the *CRY2*^*S401F*^ and *CRY2*^*D393G*^ haplotypes were found in *R. palustris* accessions (**a**). Letters indicate significant differences as determined by ordinary one-way ANOVA. **c** Y2H assays showing the interactions between different versions of CRY2 and CIB1. Transformed yeast cells were grown on SD/-Leu/-Trp/-His plates supplemented with 5–25 mM 3-amino-1,2,4-triazole (3-AT) under dark or light conditions. AD, GAL4 activation domain; BD, GAL4 DNA binding domain. **d** A close-up view of the CRY2 structure (PBD ID 6M79). The FAD molecule and mutated residues are displayed as sticks and colored in yellow and violet, respectively. Helices α15–α17 with mutated residues are colored in light blue. **e** Proposed model for the evolution of weed ruderality in Brassicaceae. Three genotypes (*FLC CRY2*, *flc CRY2*, and *flc CRY2*^*W374M*^) are shown. The source data underlying Fig. 6a, b are provided as a Source Data file.

A short life-cycle is a typical feature of ruderal plants. We, therefore, speculate that the dominant *CRY2* mutation may serve as a conserved evolutionary driver of the early-flowering phenotype of ruderals. To test this, we collected 13 accessions of *R. palustris*, another common Brassicaceae ruderal weed species in China (Supplementary Data 7)^67^. Flowering time measurements revealed that all the accessions except Zhangdy334 did not require vernalization to flower early. Among 12 non-vernalization-requiring accessions, four accessions flowered at nearly the same time under LD and SD conditions, whereas the other eight accessions did not flower after one year under SD conditions (Fig. 6a). Interestingly, DNA sequencing revealed that all the photoperiod-insensitive *R. palustris* accessions harbored either Phenylalanine (F) instead of Serine (S) at position 401 (CRY2^S401F^) or Glycine (G) instead of Aspartate (D) at position 393 (CRY2^D393G^) in CRY2 (Fig. 6a). Notably, Y2H and transgenic plant studies revealed that both CRY2^S401F^ and CRY2^D393G^ are constitutively active, just like CRY2^W374M^, CRY2^V367M^ and CRY2^V360M^ (Fig. 6b, c). Consistent with this finding, structure analysis indicated that S401 and A393 are located in helix 17 near the chromophore FAD binding site (Fig. 6d)^68,69^. Thus, these findings show that the dominant mutations in CRY2, although occurring at different residues from those in *C. occulta*, also contribute to the weedy ephemeral strategy of *R. palustris*.

## Discussion

Our results suggest a common evolutionary trajectory underlying a short life-cycle in the Brassicaceae ruderal weeds. The loss of the vernalization requirement (through the mutation of *FLC* shown here) and a subsequent dominant mutation in the blue-light receptor gene *CRY2* enable plants to maximize the number of seeds that enter the seed bank prior to disturbance, thereby increasing the number of offspring in environments with a high frequency of disturbance (Fig. 6e). This conclusion is further supported by the findings that pop3, a widely spreading population that carries the *CRY2*^*W374M*^ mutation, has evolved from pop2 and exhibits the lowest genetic diversity (Fig. 1). Notably, the emergence of the *CRY2*^*V367M*^ mutation in some pop2 accessions likely recapitulated this ancient evolutionary process (Fig. 1c).

It should be emphasized that early flowering is necessary but not sufficient for the establishment of the ruderal growth habit. While there are many genes involved in flowering time control, why is *CRY2* preferentially selected in this context? This question may be addressed from following two aspects: First, many weed species are polyploids. Our population genomics study pinpointed the evolutionary advantage of the dominant *CRY2* mutation; a constitutively active CRY2 protein facilitates the rapid spread of these accessions within a local population, even in a polyploid background. Second, in contrast to other flowering time regulators, CRY2 exerts pleiotropic effects on plant development and physiology^70^. Growing evidence has shown that CRY2, in addition to flowering time, regulates shade avoidance^71^, temperature response^72,73^, and plant growth^74–78^. Therefore, it is highly possible that diverse biological pathways governed by blue-light signaling also contribute to the evolution of weed ruderality, albeit their precise molecular mechanisms await further investigations.

Recent studies have highlighted the importance of convergent evolution in the evolution of agricultural weeds^3^. For example, genome sequencing of 163 waterhemp (*Amaranthus tuberculatus*) individuals from Canada and the United States revealed that widespread herbicide resistance likely arose from both convergent adaptation and hybridization^79^. Similarly, although Chinese weedy rice was de-domesticated independently multiple times, the genomic signature for convergent evolution in different weedy types is evident^14,15^. Our results now provide compelling evidence that convergent evolution could also occur in weedy species within the same genus. Both *C. occulta* and *R. palustris* harbor dominant mutations in *CRY2*. Notably, the constitutively active form CRY2 renders plants able to deposit seeds into the soil seed bank earlier, thereby escaping human disturbance. This finding is consistent with the idea that convergent evolution often arises when different species occupy similar ecological niches and adapt in similar ways under similar selective pressures. Interestingly, a recent study revealed that weedy rice could benefit from earlier flowering because it shortens the entire growth period as well^80^. While the causal gene remains to be identified^81^, all these results clearly demonstrate a critical and common role of a short life-cycle in weed ruderality.

Our results suggest that selective pressure (in this case, artificial disturbance) has a profound impact on shaping local population composition in weeds. While the wild-type *C. occulta* plants are dominant under undisturbed conditions, individuals carrying the *CRY2*^*W374M*^ mutation will quickly dominate the whole population in high-disturbance environments, even when they are originally present at low frequency. Thus, the adaptive advantage conferred by the *CRY2*^*W374M*^ mutation is niche-dependent and needs to be maintained by artificial disturbance. Importantly, this observation could explain why some individuals harboring the *FLC* or *CRY2* mutation still flower late (Fig. 5a; Supplementary Data 1). It is likely that these plants evolved at a second or third genetic locus to counter the effects of early-flowering caused by the *FLC* or *CRY2* mutation, thereby regaining an advantage under undisturbed conditions. Such a scenario has been observed in the evolution of weedy rice through de-domestication, where weedy rice varieties usually display a suite of traits that are intermediate between wild and cultivated rice^13,14,18,28,82^.

Humans and weeds share a long co-evolutionary history. Harvest weed seed control (HWSC) is one of the most popular non-chemical weed management techniques to limit weed reproduction and thereby give effective control of herbicide-resistant weed biotypes^2,83^. Our work highlights recent concern that the long-term application of HWSC will drive weed evolution in ways that will avoid the combine seed mills, with the obvious one being a trend toward early deposition of seeds into the soil seed bank^83^. For instance, recent studies in *Raphanus raphanistrum* have uncovered a directional selection for early flowering owing to HWSC selection pressure^84–86^. Therefore, exposing few individuals to the selection pressure, thereby maintaining low weed density, is needed for truly sustainable weed management.

While this study suggests the crucial role of a short life-cycle in weed ruderality, we cannot exclude the possibility that other functional properties and genes contribute to the adaption of pop3 to high-disturbance environments. Future research should dissect whether better adaption to nutrient-rich soil and enhanced tolerance to herbivory insects and pathogens are also involved in the evolution of weed ruderality in Brassicaceae. The past 5 years have witnessed great progress in sequencing weed genomes, owing to a continued reduction in costs for DNA sequencing and the recognition of the importance of studying human-crop-weed systems for addressing basic science questions related to plant adaption, evolution, and ecology^3,5,8,25^. We envision that the implementation of the Earth BioGenome project^87,88^, a joint effort of the International Weed Genomics Consortium^29^, large-scale phenotyping, and field experiments will help us to understand the genetic basis underlying diverse plant life-history strategies and agricultural weed syndrome in the near future.

## Methods

### Sampling

The *C. occulta* accessions used in this study were collected from China, Thailand, and Japan^30,33^. Among them, 26 accessions were ordered from the Germplasm Bank of Wild Species (http://www.genobank.org), and one accession was ordered from the Sendai *Arabidopsis* Seed Stock Center (https://sassc.epd.brc.riken.jp). Briefly, we ordered all the Cardamine accessions from the Germplasm Bank of Wild Species and verified the taxonomy of these plants by phenotypic analysis (Supplementary Fig. 1a), flow cytometric assay (Supplementary Fig. 1b), and genome re-sequencing (Supplementary Data 1). It should be noted that the paper describing the taxonomy of *C. occulta* has not been published^30^ when the Cardamine seeds from the Germplasm Bank of Wild Species were collected from 2005 to 2013. As a result, only the verified octoploid *C. occulta* accessions were used in this study.

The *R. palustris* accessions were ordered from the Germplasm Bank of Wild Species and the Germplasm Resources Information Network (https://www.ars-grin.gov). *C. scutata* and *C. kokaiensis* plants were collected in Japan (Shirakawa) and Shanghai (Minhang district). Detailed sample information can be found in Supplementary Data 1. The distribution maps showing sample location, disturbance category, and subgroup information were generated by the R package ggplot2.

### Plant materials and growth conditions

The *C. occulta*, *A. thaliana*, and *R. palustris* accession plants were grown on soil at 21 °C in the growth chambers under LD (16-h light/8-h dark) or SD (8-h light/16-h dark) conditions. For vernalization treatment, the seedlings with fully expanded cotyledons were grown in a 4 °C growth chamber under SD conditions for two months, and then returned to 21 °C LD conditions. The *A. thaliana* accession Columbia-0 (Col-0) was used as wild-type. The *cry2-*1, *FRI*^*SF2*^ *FLC* and *FRI*^*SF2*^ *flc-*3 mutants have been reported^55,89,90^.

### Genome size estimation

The mapping rate of all the *C. occulta* accessions was above 83.7% (Supplementary Data 1). Illumina re-sequencing reads of all the *C. occulta* accessions were assembled using SPAdes (v3.13.0) with kmer 77^91^. To estimate the genome size by flow cytometry assay, plant homogenates were prepared as described with modifications^92^. Briefly, four rosette leaves were chopped in Galbraith’s buffer and stained with 4,6-diamidino-2-phenylindole (DAPI, AAT Bioquest, Cat No./ID: 28718903)^93^. A minimum of 10,000 nuclei for each biological replicate were analyzed on a flow cytometer (Beckman Coulter, MoFlo XDP) equipped with a 355 nm laser. The histograms were visualized and analyzed using the FlowJo software (https://www.flowjo.com). Three independent replicates were analyzed. The Yunnan accession was used as an external standard to estimate the ploidy and genome sizes of other accessions^94^. The Yunnan accession also served as an internal standard to determine whether representative accessions of other subgroups have the same genome size (Supplementary Fig. 1b). The estimated genome sizes of all the *C. occulta* accessions by de novo assembly and flow cytometry assay can be found in Supplementary Data 1.

### Genome sequencing and assembly

Total DNAs for genome sequencing were extracted from young leaves of the Yunnan accession. DNA library was constructed from the high-quality genomic DNA prepared from a single plant using the SQK-LSK109 kit following the standard protocol of ONT. The PromethION platform (R9.4.1; FLO-PRO002; Biomarker Technologies) was used to generate Nanopore data (binary fast5 format). The raw data was subjected to base calling using the Guppy software from the MinKNOW package and additional quality-control step was performed to remove sequencing adapter and reads with low quality and/or short length (<2000 bp). The Hi-C library^95^ was prepared using restriction enzyme HindIII according to the instruction of NextOmics Technologies Company and sequenced on the Illumina Hiseq platform (Illumina, San Diego, CA, USA). The DNA extracts used for whole-genome re-sequencing were sequenced using Illumina NovaSeq platform at ~100× genomic coverage with 150-bp read length and 300 − 500 bp insert size.

We de novo assembled ONT long reads into contigs using Canu assembler^38^ with the settings ‘minReadLength=5000, minOverlapLength=2500, -nanopore-corrected’. To correct base errors, two rounds of polishing were then applied to the raw contigs using NextPolish^39^. The resulting polished contigs were assembled into the pseudo-chromosomes using the 3D-DNA pipeline^96^ and ALLHiC^40^. 3D-DNA pipeline was used to map the Hi-C reads into contigs and split the mis-join contigs based on the Hi-C linking information. ALLHiC was used to scaffold the corrected-contigs into the pseudo-chromosomes based on the proximity-guided assembly. Collinearity detection was performed with WGDI^97^. The assembled genome was visualized by Circos^98^.

### Repeat annotation

We combined RepeatModeler (http://www.repeatmasker.org/RepeatModeler/) and RepeatMasker (http://www.repeatmasker.org/RepeatMasker/) to annotate repeated sequences in the *C. occulta* genome. RepeatModeler was used to generate de novo transposable element (TE) sequences. The custom TE libraries were imported into RepeatMasker to identify and cluster repetitive elements.

### Gene annotation

To annotate protein-coding genes, we developed an automatic annotation pipeline by iteratively calling MAKER^99^. In the first round, we combined the transcripts assembled from the RNA-seq datasets of three tissues (root, leaf, and flower) using STAR^100^ and StringTie^101^. The homologous proteins from Swiss-Prot were used to train the SNAP HMM model. In the second and third rounds, we updated the SNAP HMM model with the transcripts and homologous proteins. In the fourth round, we selected high-quality gene models predicted by the SNAP to train AUGUSTUS. Finally, we used the MAKER to integrate ab initio gene predictors (SNAP^102^ and AUGUSTUS^103^), transcripts, and homologous proteins to identify and annotate protein-coding genes. Gene structures were visualized in Apollo^104^ along with assembled transcripts and homologs.

### Re-sequencing

Young leaves from a single plant of each accession were harvested. Genomic DNAs were prepared with the Super Plant Genomic DNA Kit (Tiangen, Cat No./ID: 4992879) according to the manufacturer’s instructions. Library construction and re-sequencing were performed on an Illumina HiSeq 4000 Platform (PE150) (Novogene, Beijing, China), with an average coverage depth of approximately 17.75× for each accession (Supplementary Data 1).

### Variant calling and annotation

A total of 1.1 trillion base pairs of raw reads were filtered by fastp (version 0.20.0) using default parameters^105^, and aligned to the Yunnan reference genome (version 1.0) using BWA-MEM with default parameters^106^. SNP calling was performed according to the GATK best practice^107^. The alignment bam files were then sorted and PCR duplicates were marked by MarkDuplictes. HaplotypeCaller (GATK version 4.1.2.0) was run on each bam file in a genomic variant call format mode^108^. The GVCF files from 82 accessions were consolidated into a single GVCF file, from which SNPs were identified using a joint calling approach. To obtain high-quality SNPs, we initially used the GATK hard filter to filter the merged VCF data with the options (QD < 2.0|| MQ < 40.0||FS > 60.0||SOR > 3.0|| MQRankSum < −12.5|| ReadPosRankSum < −8.0). Biallelic SNPs with an integrity rate greater than 0.9, a minor allele frequency (MAF) greater than 0.05, and a heterozygous site ratio less than 0.2 were filtered, resulting in a set of 4.7 million high-quality SNPs which were subsequently used for population analyses. We annotated the variants using SnpEff (version 4.3)^109^, based on the gene annotation file of the *C. occulta* genome.

### Neighbor-joining tree and population structure analysis

We constructed a neighbor-joining (NJ) tree using MEGA X^110^ with 1000 bootstraps. The tree layout was generated using EvolView^111^. PCA was performed using PLINK (version 1.9)^112^. The population structure was analyzed with the cluster number k ranging from 2 to 7 by ADMIXTURE (version 1.3.0)^113^, using SNPs filtered by PLINK with parameters “–indep-pairwise 50 10 0.2”. The output result for *k* = 3 was visualized using the R package pophelper^114^. Linkage disequilibrium decay was calculated by pairwise correlation coefficient (*r*^2^) for all SNP pairs within 100 kb, using a heterozygous site ratio less than 0.02 SNP set, and plotted by PopLDdecay (Version 3.40)^115^. Nucleotide diversity (*π*) values were calculated using VCFtools (Version 0.1.17)^116^ with a window size of 50 kb and a step size of 20 kb.

The genotype of *C. occulta*, which was used to polarize SNPs as either ancestral or derived, was determined by the reference genome accession (Yunnan) and the other two accessions (Pingshui and HANGYY8053) from pop1 and pop2 respectively. The derived allele frequencies of three subgroups were calculated by VCFtools^116^.

### Demography inference

The demographic history of *C. occulta* was inferred using SMC++(Version 1.15.4)^41^, which could simultaneously analyze a large number of samples and is powerful for recovering population history at short timescales. Since *C. occulta* is self-fertilized, only the homozygous SNP sites without missing data were used. We randomly selected 14 and 30 individuals from pop2 and pop3, respectively, and created pseudodiploids by combining haplotypes from random pairs of these individuals from the same subgroups^117^. SMC++ split model was then run on all the pseudodiploids using default parameters, with the masking file created by RepeatMasker (Version 4.1.1) (https://www.repeatmasker.org/). The mutation rate was assumed as *μ* = 7.1 × 10^−9^ mutations × bp^−1^ × generation^−1^ as in *A. thaliana*^117^.

### Identification of differentiation signals

To identify candidate regions potentially associated with adaptation, fixation statistics (*F*_ST_) between pop2 and pop3 were calculated using VCFtools (Version 0.1.17) in a 50 kb sliding window with a step size of 20 kb. Sliding windows with top 5% *F*_ST_ values of genome-wide *F*_ST_ values were selected and assigned as significantly different windows. Overlapping significance windows were merged into fragments, which were considered highly diverged regions across pop2 and pop3. The annotated genes residing in these regions were considered candidate adaptive genes. We then used the BLAST (Version 2.10.1) algorithm to identify the orthologs of these candidate genes in *A. thaliana*. Only the best hits from the BLAST results were retained and used for GO enrichment analysis. GO enrichment analysis was performed using org.At.tair.db (Version 3.10.0) (https://bioconductor.org/packages/release/data/annotation/html/org.At.tair.db.html) and clusterProfiler (Version 3.14.0)^118^. GO terms with corrected *P* values <0.05 were considered significantly enriched and sorted in ascending order of corrected *P* values (Supplementary Data 4). The top eight GO terms were showed in Fig. 2c. GO analysis was further confirmed by SNP2GO^42^. The enriched GO terms were summarized and visualized using the R package simplifyEnrichment based on semantic similarity (Supplementary Figs. 2a,b)^119^.

### Bulk segregation analysis

To identify the causal mutations responsible for the photoperiod sensitivity variation, the Pudong (pop3, early-flowering in SD) and Yunnan (pop1, late-flowering in SD) accessions were used to construct an F_2_ population. The flowering times of the 311 F_2_ individuals segregated under SD conditions. The early-flowering and late-flowering DNA pools were constructed by mixing equal amounts of DNAs from 50 early-flowering F_2_ individuals and 49 late-flowering F_2_ individuals, respectively. The bulked DNA samples and two parental DNA samples were subjected to whole-genome sequencing and variation calling using the same methods as used for the population re-sequencing. Approximately 33- to 45-fold genome sequences for each parent and bulk samples were generated. SNPs between two parental genomes with a total depth from 15 to 115 were calculated for a ΔSNP index using R package QTLseqr^120^. The candidate genes were determined in the genomic regions with ΔSNP index above the threshold at the 99% confidence intervals.

To identify the causal mutation(s) responsible for the loss of vernalization requirement in pop3, we generated the F_2_ population derived from a cross between the Pudong (pop3, early-flowering in LD without vernalization) and HANGYY8055 (pop2, early-flowering in LD in response to vernalization) accessions. The flowering times of 358 F_2_ individuals were segregated under LD conditions. The early-flowering and late-flowering DNA pools were constructed by 43 early-flowering F_2_ individuals and 46 late-flowering F2 individuals, respectively. Approximately 39- to 41-fold genome sequences for each parent and bulk sample were generated and aligned to the alternative Pudong accession reference sequence, which was generated by FastaAlternateReferenceMaker (GATK version 4.1.2.0). The SNPs with total depth from 10 to 100 were calculated for a ΔSNP index using the R package QTLseqr.

### RNA-seq analysis

The Yunnan, Pudong, and HANGYY8055 accessions were grown in a growth chamber under LD conditions. We performed three biological replicates. For each biological replicate, we harvested the third fully expanded leaves from at least six individuals at ZT16. Total RNAs were extracted with the Trizol reagent (ThermoFisher, Cat No./ID: 15596018). Library construction and sequencing were performed on an Illumina HiSeq 4000 Platform (Novogene, Beijing, China). Raw reads were filtered with fastp (version 0.20.0), and aligned to the *C. occulta* Yunnan reference genome (version 1.0) using hisat2 (Version 2.1.0)^121^ with default parameters. The resulting sam file containing mapped reads were converted to the bam format, sorted, and indexed using SAMtools (Version 1.9)^122^. Gene counts were called from the resulting bam files using featureCounts (Version 1.6.2)^123^, with the parameter “-p”, and differential expression analysis was conducted using the R package DESeq2 (Version 3.10)^124^.

### Constructs and generation of transgenic plants

The primer sequences and constructs generated in this study are given in Supplementary Data 8 and 9. For the Y2H constructs, the cDNAs of *AtCRY2*, *AtCIB1*, *CoCRY2*, *CoSPA1*, and *CoCIB1* were PCR-amplified and cloned into the pGBKT7 or pGADT7 vectors (Clontech). The mutated forms of *CRY2* (*CRY2*^*W374M*^, *CRY2*^*V367M*^, *CRY2*^*S401F*^, *CRY2*^*D393G*^, and *CRY2*^*V360M*^) were generated by site-directed mutagenesis using *AtCRY2* as the template. The cDNA of *CoCRY2*^*W374M*^ (Chr 1 A) was PCR-amplified from the Pudong accession, and used as a template to generate *CoCRY2*^*WT*^ using a site-directed mutagenesis approach. Since *C. occulta* is an octoploid, we only selected a representative copy of *CoSPA1* or *CoCIB1* from the Pudong accession for the Y2H assay.

To generate *pCoCRY2::CoCRY2*^*W374M*^ and *pCoCRY2::CoCRY2* constructs, the genomic region of *CoCRY2* (Chr 1 A), which includes a 1.8 kb upstream and an 0.4 kb downstream fragments, was PCR-amplified from the Pudong accession. The cDNA fragments of *CoCRY2*^*W374M*^ and *CoCRY2* were fused with 6xMyc tag at the N-terminal, and cloned into the binary construct LZ118 or LZ120.

The amiRNA construct *35* *S::amiR-CoCRY2* was designed and generated by the WMD3 server (http://wmd3.weigelworld.org/cgi-bin/webapp.cgi)^125,126^. The CaMV *35* *S* promoter was used to drive *amiR-CoCRY2* expression.

To generate the *pAtCRY2::6×Myc-AtCRY2* series constructs, the wild-type or mutated *AtCRY2* cDNA fragments were fused with 6×Myc tag at the N-terminal, cloned into the binary vector LZ100, which harbors a 3.0 kb upstream and a 2.1 kb downstream fragment of *AtCRY2*.

To generate the *AtFLC* and *AtFLC_truncated* constructs, the 11.9 kb wild-type or mutated *AtFLC* genomic fragments, which include 3.4 kb upstream and an 2.8 kb downstream fragments, were cloned into the binary vector AA00.

The binary constructs were delivered into *Agrobacterium tumefacien*s strain GV3101 (pMP90) by the freeze-thaw method. Transgenic plants were generated by the floral dipping method^127^ for *A. thaliana*, or by the floral vacuum infiltration method for *C. occulta*^128^. The transgenic plants were screened with 0.05% glufosinate (Basta) on soil.

### Flowering time measurement

To measure flowering time, the total number of leaves when plants started to bolt was counted. The SD/LD ratio was calculated by dividing the median number of total leaves when plants started to bolt in SD by the median number of total leaves when plants started to bolt in LD. The SD/LD ratio was then used as an indicator of photoperiod sensitivity (Figs. 2d, 5a; Supplementary Fig. 2c). The plants with vernalization requirement are the plants flowering with a total leaf number greater than 25 under long-day conditions but less than or close to 10 after vernalization treatment.

### Y2H assay

Plasmids were transformed into yeast strain AH109 (Clontech) by the LiAc/SS Carrier DNA/PEG method^129^. The transformants were selected on SD -Leu-Trp plates. The interactions were tested on SD -Leu-Trp-His (SD -LWH) or SD -Ade-Leu-Trp-His (SD -ALWH) plates supplemented with 5-25 mM 3-AT. At least six individual clones for each combination were analyzed. For the light treatment, red or blue-light was provided by red or blue-light-emitting diodes (LEDs) respectively, with light intensities of 40 µmol m^−2^ s^−1^.

### Expression analysis

Total RNAs were extracted using the Trizol reagent (ThermoFisher, Cat No./ID: 15596018). The RNAs were treated with DNase I (ThermoFisher, Cat No./ID: EN0521) and subjected to the 1st strand complementary DNA (cDNA) synthesis using the RevertAid First Strand cDNA Synthesis Kit (ThermoFisher, Cat No./ID: K1622) with oligo (dT) primer. The gene expression levels were determined by RT-qPCR using TB Green Premix Ex Taq II (Takara, Cat No./ID: RR820B) with ROX Reference Dye II. The relative gene expression levels were calculated by 2^−ΔΔCt^ values and normalized using *CoSAND* as the reference gene^130,131^. The primer sequences are given in Supplementary Data 8.

### CAPS

Cleaved amplified polymorphic sequences (CAPS) were used to discriminate the Pudong and Yunnan accessions. Since the *CRY2*^*W374M*^ mutation exists only in Pudong but not in Yunnan, the individuals carrying this mutation were identified as Pudong, while those without this mutation were identified as Yunnan. The *CRY2*^*W374M*^ allele is a mutation from TG to AT, creating a *Nco*I recognition site. For the CAPS assay, the primers in Supplementary Data 8 were used to amplify a mapped DNA sequence. The amplified fragment from Pudong contains the *Nco*I recognition site and can be cleaved into two additional fragments. When fractionated by agarose, the PCR products digested by *Nco*I will give readily distinguishable patterns.

### Statistical analyses

GO enrichment analysis was performed using the R package clusterProfiler. The *P* value was calculated by one-sided Fisher’s exact test and adjusted for multiple comparisons using the Benjamini and Hochberg methods. GO terms with corrected *P* values <0.05 were considered significantly enriched.

For phenotypic evaluation, at least eleven individual plants were analyzed for each accession and the exact number of individuals (*n*) is indicated in the figures. Significance levels of differences were calculated by one-way ANOVA with GraphPad Prism 8 (version. 8.0.1).

For RNA-seq analysis, normalized counts and adjusted *P* values were both analyzed by DESeq2. The *P* values attained by the Wald test were corrected for multiple comparisons using the Benjamini and Hochberg methods. One, two, and three stars (*) in the figures represent *P* values <0.05, <0.01, and <0.001, respectively.

### Reporting summary

Further information on research design is available in the Nature Portfolio Reporting Summary linked to this article.

## Supplementary information

Supplementary Information

Description of Additional Supplementary Files

Supplementary Data 1

Supplementary Data 2

Supplementary Data 3

Supplementary Data 4

Supplementary Data 5

Supplementary Data 6

Supplementary Data 7

Supplementary Data 8

Supplementary Data 9

Reporting Summary

## Data Availability

The genome sequence of *C. occulta* and RNA-seq data generated in this study have been deposited in NCBI under accession code PRJNA846126. Source data are provided with this paper.

## Source data

Source Data
