# Supplementary material for: Common evolutionary trajectory of short life-cycle in Brassicaceae ruderal weeds
Source: Nat Commun. 2023 Jan 18;14:290. doi: 10.1038/s41467-023-35966-7 (PMC9849336; doi:10.1038/s41467-023-35966-7)
Supplement: Supplementary file 1 — Supplementary Information [file 41467_2023_35966_MOESM1_ESM.pdf]

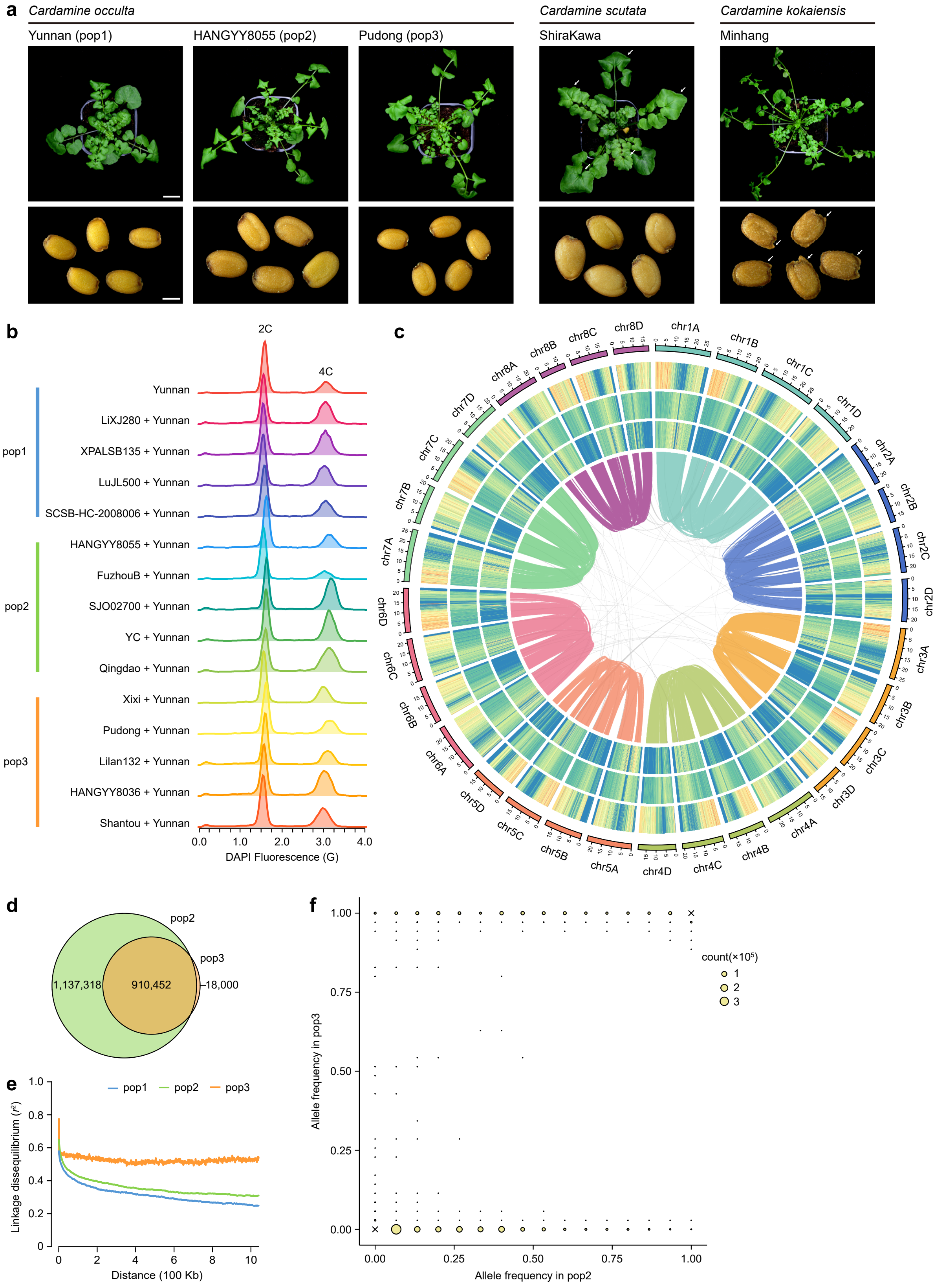

Supplementary Fig. 1 | Additional data related to genome assembly and population analysis of *C. occulta* plants

a, Morphological differences between *C. occulta* and its tetraploid progenitors, *C. scutata* (accession Shirakawa) and *C. kokaiensis* (accession Minhang). One representative accession for each *C. occulta* subgroup is shown. For *C. scutata*, their terminal leaflets (white arrows) are longer and wider than lateral leaflets, and angles of the apexes of leaflets are also more obtuse. These characters separate *C. scutata* from *C. occulta* [32]. For *C. kokaiensis*, they are clearly distinguishable by narrow wings (white arrows) all around the seeds [32]. Scale bar, 2 cm for seedlings and 2 mm for seeds. **b,** Histograms of *C. occulta* nuclear DAPI-intensity. Five representative accessions for each subgroup were analyzed. The first and second peaks in the histogram represent 2C and 4C nuclei respectively. The Yunnan accession was analyzed alone as a control and served as an internal standard for the accessions of other subgroups in other histograms. Note that all the accessions have the same genome size as Yunnan. Histograms are representatives of three independent biological replicates. **c,** Circos display of important features of the assembled *C. occulta* genome (Yunnan accession). The circles (from outermost to innermost) represent chromosome names and sizes with scale mark labeling each 5 Mb, gene density, transposable element density, SNP density, and links between syntenic genes. Genomic features were calculated using 50 kb non-overlap window. Red and blue colors indicate high and low density respectively. **d,** Venn diagram showing the numbers of specific and shared SNPs between pop2 and pop3. **e,** Decay of linkage disequilibrium, indicated as r^2, in three subgroups. **f,** Allele frequency distribution of SNPs in pop2 and pop3. The cross indicates that the number of SNPs is not shown.

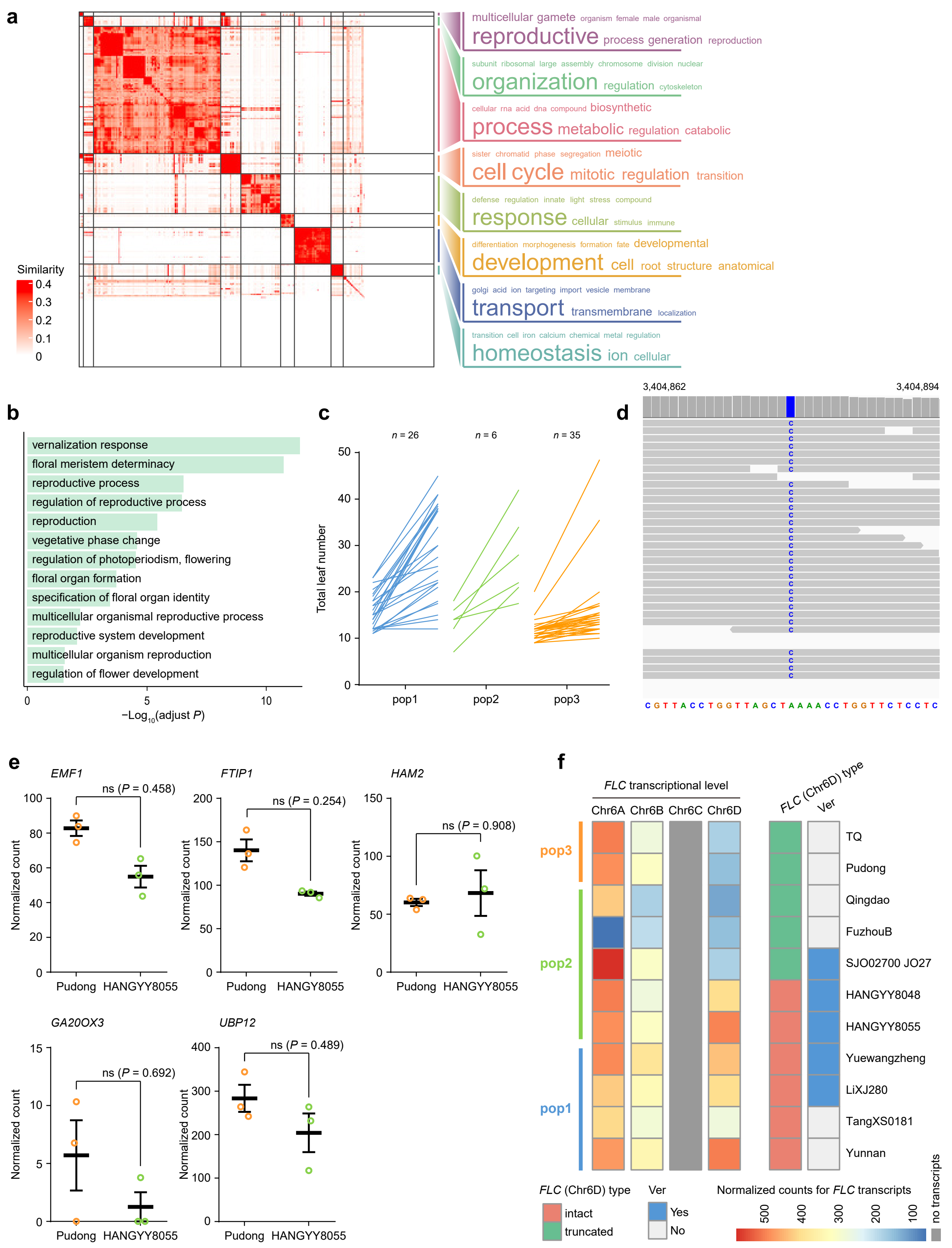

Supplementary Fig. 2 | GO term analyses by SNP2GO and additional data supporting *FLC* as the gene responsible for the loss of vernalization requirement in pop3

a, Clustered (binary cut) semantic similarity matrix of biological process GO terms for all the genes harboring candidate SNPs within the top 5% F_{ST} regions. Word cloud annotations on the right side summarize the features with keywords in each GO cluster. The color shade reflects the similarity of the pathway enrichment, while different dimensions represent the size of the enrichment. **b,** GO term analyses of the SNPs within the top 5% F_{ST} regions calculated by SNP2GO. The 15 selected enriched GO biological processes are indicated. **c,** Flowering time of *C. occulta* accessions. Each line represents an accession. The median number of total leaves when plants started to bolt in LD (the left end of each line) and SD (the right end of each line) were shown. The accessions were sorted by subgroups. The number of the accessions in each subgroup is given on the top. Please note that most pop3 accessions flowered early in SD. See also Fig. 2d. **d,** Mapping interval at the *FLC* (Chr 6D) locus. The colored reference sequence is shown at the bottom. The gray boxes represent the same sequence as the reference and the mutation site (T to G mutation) is labelled in blue. **e,** Analysis of the transcript levels of the candidate genes in the Pudong and HANGYY8055 accessions by RNA-seq. Normalized counts and adjusted *P* values were both analyzed by DESeq2. The *P* values attained by the two-sided Wald test were corrected for multiple testing using the Benjamini and Hochberg method. Data are mean ± s.e. of three biological replicates. Each open circle represents a biological replicate. ns, not significant. The centers of the error bars represent the mean values. **f,** Analysis of the genotype and transcript levels of *FLC*. Eleven representative accessions from pop1, pop2 and pop3 were shown. The vernalization requirement (Ver) and genotype of *FLC* on Chr 6D (intact or truncated) was given. Please note that the *FLC* copy on Chr 6C lacked exons 2, 3, and 4 in all the *C. occulta* accessions and no full-length transcripts were counted. Normalized counts were analyzed by DESeq2.

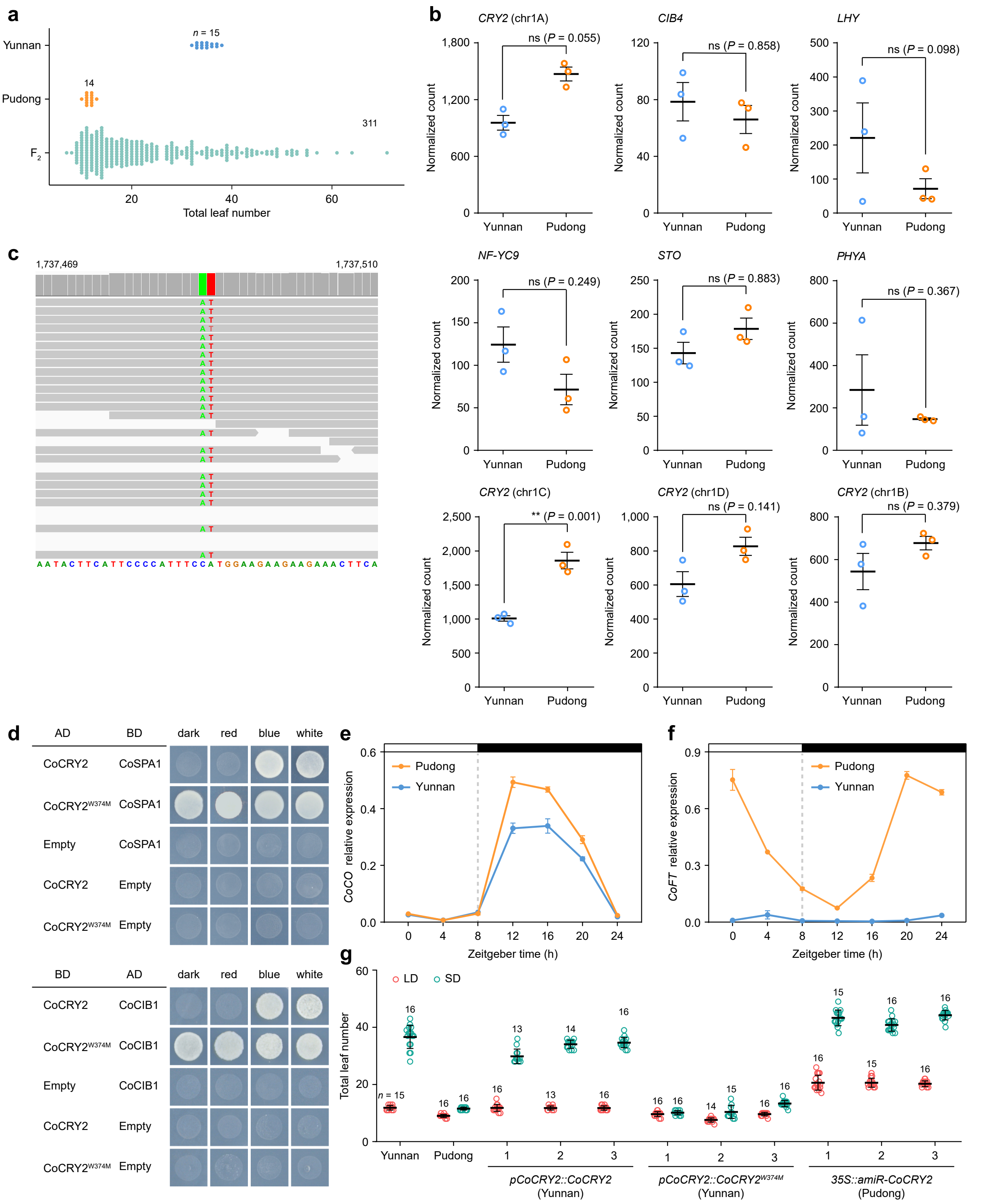

Supplementary Fig. 3 | Additional data supporting *CRY2* as the gene responsible for the early-flowering phenotype of the Pudong accession

a, Flowering time of the Yunnan and Pudong accessions and plants in the Yunnan × Pudong F_2 segregating population under SD conditions. Plants were grown in a growth chamber. The total number of leaves when plants started to bolt was counted. The number of plants examined is given. **b,** Analysis

of candidate gene transcript level by RNA-seq in *C. occulta* accessions Yunnan and Pudong. The expression levels of *CRY2* on Chr 1B, Chr 1C, and Chr 1D are shown. Normalized counts and adjusted *P* value were both analyzed by DESeq2. The *P* values attained by the two-sided Wald test were corrected for multiple testing using the Benjamini and Hochberg method. Data are means ± s.e. and each open circle represents a biological replicate. ns, not significant. The centers of the error bars represent the mean values. **c,** Mapping interval at the *CRY2* locus of the Pudong accession. The colored reference sequence is shown at the bottom. The gray boxes represent the same sequences as the reference and the mutation sites are labelled in green or red. **d,** Y2H assays showing the interactions between CoCRY2 or CoCRY2^{W374M} and CoSPA1 or CoCIB1. Transformed yeast cells were grown on a SD/-Leu/-Trp/-His plate supplemented with 5-25 mM 3-AT under different light conditions. AD, GAL4 activation domain; BD, GAL4 DNA binding domain. **e,f,** Temporal expression pattern of *CO* (**e**) and *FT* (**f**) in the Yunnan and Pudong accessions in SD. Eight plants were harvested at 4-hour interval over a 24 hour period. Sampling time is shown in hours as ZT. Error bars denote s.d. of three technical replicates. Four biological replicates were performed with consistent expression trend. The centers of the error bars represent the mean values. **g,** Flowering time of *C. occulta* accessions and three independent transgenic lines under LD and SD conditions. The total number of leaves when plants started to bolt was counted. The number of examined plants (*n*) is given. Error bars denote s.d. The centers of the error bars represent the mean values.

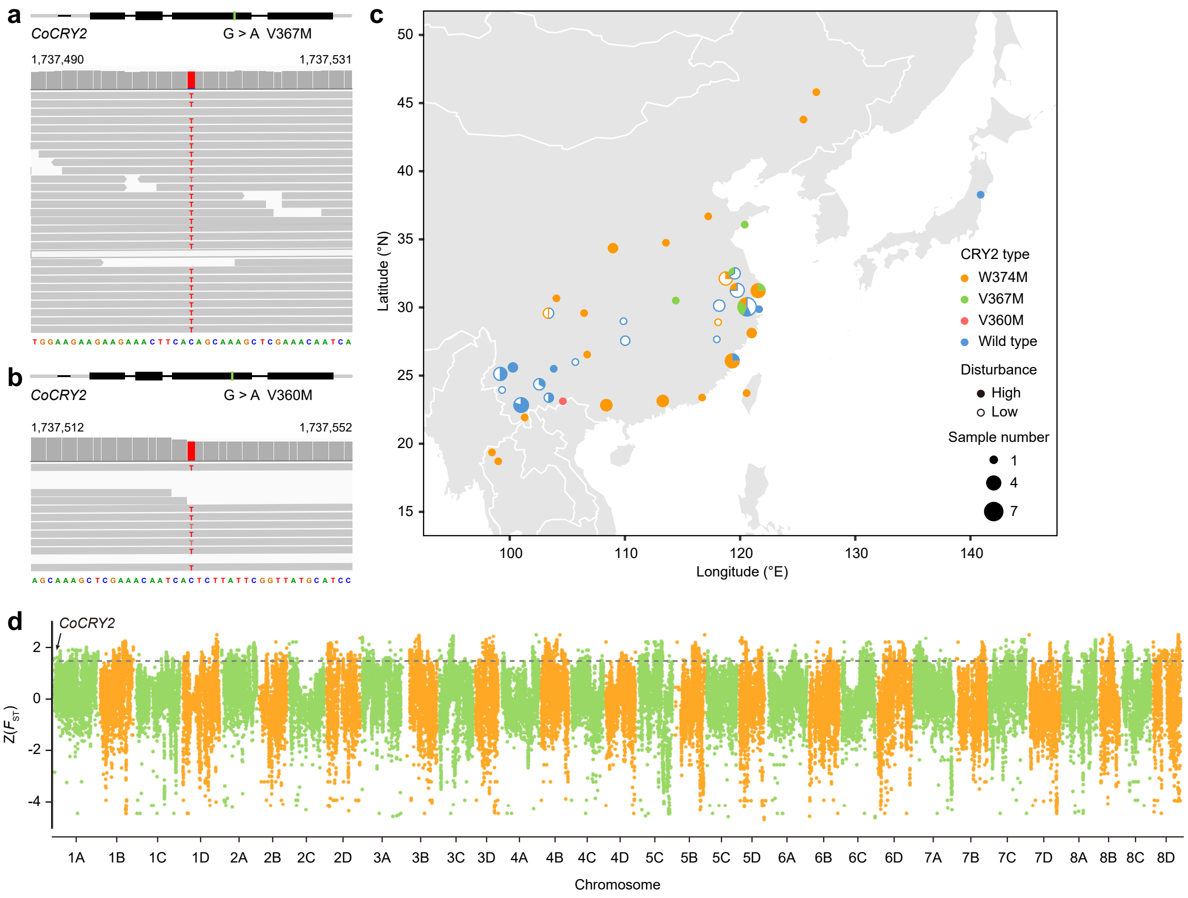

Supplementary Fig. 4 | Additional data supporting *CRY2* as the gene responsible for the early-flowering phenotype of the Pudong accession

a,b, The gene structure of *CRY2* and mapping interval at the $CRY2^{V367M}$ (**a**) or $CRY2^{V360M}$ (**b**) locus. Black boxes, gray boxes, black lines and green lines represent exons, UTRs, introns, and the location of the mutation, respectively. The colored reference sequence is shown at the bottom. The gray boxes represent the same sequences as the reference and the mutation site is marked in red. See also Fig. 1c and Supplementary Data 1. **c,** Geographic distribution of the *CRY2* allele frequency. The area of each pie represents the sample size in each site, and the colors indicate allele frequency. The filled circles and the open circles represent the high-disturbance and low-disturbance habitats, respectively. See also Supplementary Data 1. **d,** F_{ST} values of pop2 and pop3 accessions across the whole genome. The gray dashed line represents the top 5% threshold. The location of *CRY2* with significantly high F_{ST} value is indicated by a black arrow.

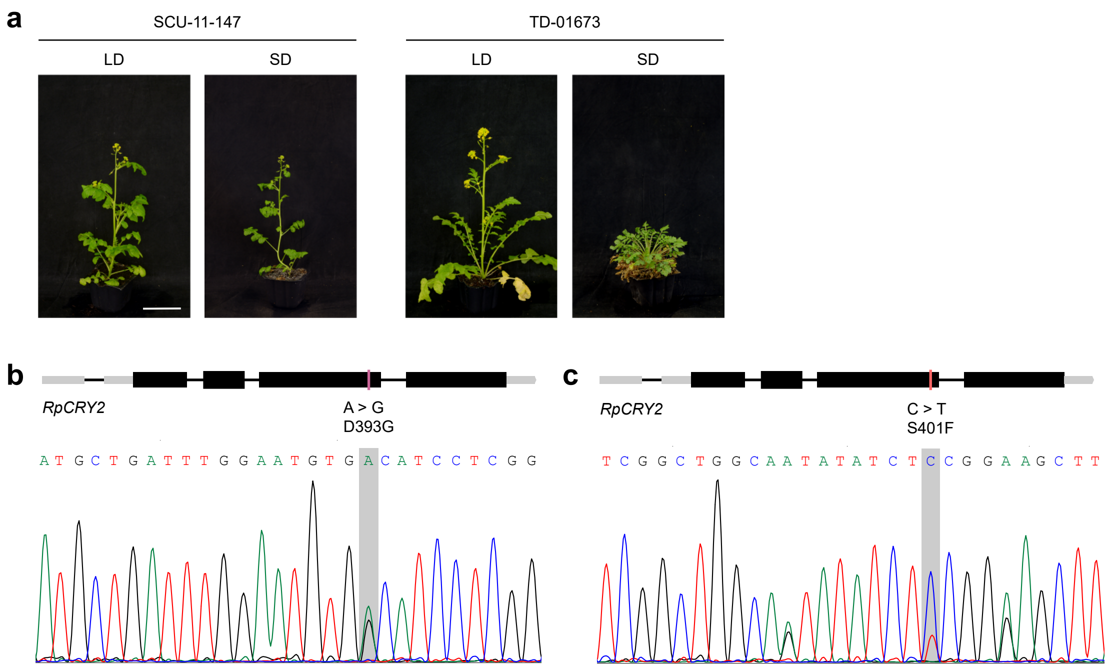

Supplementary Fig. 5 | Flowering time analysis of *R. palustris* accessions

a, Flowering phenotype of *R. palustris* accessions SCU-11-147 and TD-01673 under LD or SD conditions. Scale bar, 5 cm. Please note that the SCU-11-147 accession plants showed early-flowering phenotype in SD. **b,c,** Gene structure of *R. palustris CRY2* (*RpCRY2*) and the location of the mutations (orange lines) in *RpCRY2*. The accession that carries the RpCRY2^{S401F} allele is BianFH-433 (**b**). The RpCRY2^{D393G} allele is identified in the accessions SCU-11-147, SCSB-A-000272, and LiJ627 (**c**). Black boxes, gray boxes and black lines represent exons, UTRs and introns, respectively. The DNA sequencing results of the PCR products are shown at the bottom. The mutated nucleotides are shaded.
