# Supplementary material for: Common evolutionary trajectory of short life-cycle in Brassicaceae ruderal weeds
Source: Nat Commun. 2023 Jan 18;14:290. doi: 10.1038/s41467-023-35966-7 (PMC9849336; doi:10.1038/s41467-023-35966-7)
Supplement: Supplementary file 2 — Description of Additional Supplementary Files [file 41467_2023_35966_MOESM2_ESM.pdf]

Description of Additional Supplementary Files

File Name: Supplementary Data 1
Description: Sampling of *C. occulta* accessions

File Name: Supplementary Data 2
Description: Statistics of the *C. occulta* genome

File Name: Supplementary Data 3
Description: The SNP information of the *C. occulta* genome

File Name: Supplementary Data 4
Description: GO analysis

File Name: Supplementary Data 5
Description: Vernalization QTL region

File Name: Supplementary Data 6
Description: Photoperiod QTL region

File Name: Supplementary Data 7
Description: Sampling of *R. palustris* accessions

File Name: Supplementary Data 8
Description: Oligonucleotides used in this study

File Name: Supplementary Data 9
Description: Constructs generated in this study
